# Genotype–phenotype correlation study and mutational and hormonal analysis in a Chinese cohort with 21‐hydroxylase deficiency

**DOI:** 10.1002/mgg3.671

**Published:** 2019-04-09

**Authors:** Chao Xu, Wenyu Jia, Xiangdeng Cheng, Hui Ying, Jing Chen, Jin Xu, Qingbo Guan, Xinli Zhou, Dongmei Zheng, Guimei Li, Jiajun Zhao

**Affiliations:** ^1^ Department of Endocrinology and Metabolism Shandong Provincial Hospital affiliated to Shandong University Jinan China; ^2^ Institute of Endocrinology Shandong Academy of Clinical Medicine Jinan China; ^3^ Shandong Clinical Medical Center of Endocrinology and Metabolism Jinan China; ^4^ Department of Pediatrics Shandong Provincial Qianfoshan Hospital Jinan China; ^5^ Department of Child Health Xiamen Maternal and Child Health Hospital affiliated to Xiamen University Xiamen China; ^6^ Department of Pediatrics Shandong Provincial Hospital affiliated to Shandong University Jinan China

**Keywords:** congenital adrenal hyperplasia (CAH), *CYP21A2*, genotype–phenotype associations, mutation, steroid 21‐hydroxylase deficiency (21OHD)

## Abstract

**Background:**

Steroid 21‐hydroxylase deficiency (21OHD) is the most common enzymatic defect, but the genotype–phenotype associations have not been well established in Chinese patients. Here, a Chinese 21OHD cohort was enrolled to investigate the clinical, biochemical, and genetic characteristics of this disorder.

**Methods:**

Mutation analysis of *CYP21A2* gene, 21‐hydroxylase activity assays and in silico predictions of protein structure were performed. Genotype–phenotype associations were analyzed in both the cohort and 487 Chinese CAH patients ever reported.

**Results:**

Among the total cohort (72 patients), 47 patients (65.3%) were diagnosed as salt‐wasting (SW) phenotype, 11 (15.3%) were simple virilizing (SV) type, and 14 (19.4%) were nonclassic (NC) type. The value of FSH and LH for prediction of the SW phenotype was up to 0.862 and 0.669, respectively. Overall, the detection rate of CYP21A2 mutation was 97.9%, which revealed 25 mutations and 36 genotypes. Four novel mutations (p.L199X, p.E321del, p.H393Q, and p.L459‐P464del) were detected and induced a significantly reduced 21‐hydroxylase activity. Generally, disease severity can be predicted with the genotypes. The most common genotypes in Chinese population were I2G/I2G (12.5%), I2G/Large lesion (12.1%), I173N/I2G (10.3%), and I173N/Large lesion (9.2%). The SW form of CAH is prominent in deletion or intronic splice mutations, namely I2G/I2G (18.6%), I2G/Large lesion (17.2%) and Large lesion/Large lesion (8.6%).

**Conclusion:**

Four novel mutations were identified and a high consistency of genotype–phenotype association was found in SW CAH. Moreover, FSH and LH levels were proved to be a promising marker for predicting the severity of the disease.

## 1 INTRODUCTION

Steroid 21‐hydroxylase deficiency (21OHD, OMIM: 201910) is one of the most common inherited metabolic disorders that accounts for about 95% of the congenital adrenal hyperplasia (CAH) (El‐Maouche, Arlt, & Merke, 2017; White & Speiser, 2000). Based on the severity of disease, 21OHD can be divided into three types: the classic salt‐wasting (SW), the classic simple virilizing (SV), and the nonclassic (NC) forms. SW is the most severe type and is responsible for approximately 75% of individuals with classic 21OHD. Affected individuals lose large amounts of sodium in their urine, which can be life‐threatening in early infancy. The classic SV is characterized by elevated levels of serum 17OHP, no salt‐wasting crisis, prenatal virilization in females, and precocious pubarche in both sexes with advanced bone age (Stikkelbroeck et al., 2003). The NC form is a mild form which may show variable degrees of postnatal androgen excess but is sometimes asymptomatic (White & Speiser, 2000). In women, this form is characterized by a variety of late‐onset symptoms, including hirsutism, menstrual disturbances, and infertility (Falhammar & Nordenstrom, 2015; New, 2006). The classic forms of 21OHD occur in 1 in 15,000 newborns, while the prevalence of the nonclassic form of 21OHD is estimated to be 1 in 1,000 individuals (Pang & Shook, 1997; Speiser et al., 2018; Therrell, 2001; van der Kamp & Wit, 2004).

Inherited in an autosomal recessive pattern, 21OHD is caused by mutations in *CYP21A2* (NM_000500.9, OMIM: 613815), the gene encoding 21‐hydroxylase (P450c21) which converts progesterone (PRG) to deoxycorticosterone and 17‐hydroxyprogesterone (17OHP) to 11‐deoxycortisol, respective precursors for aldosterone and cortisol (Speiser et al., 2018). The residual 21‐hydroxylase enzyme determines the severity of the disorder (White & Speiser, 2000). Mutations lead to a complete inactivation of 21‐hydroxylase are related to the SW phenotype. Those that reduce the enzyme activity to be 1%–5% are associated with the SV phenotype, whereas those with a remaining enzyme activity ranging from 10% to 75% result in the mild NC phenotype (Krone et al., 2013; Taboas et al., 2014). Thus, the phenotype of a 21OHD fetus and the subsequent newborn can be predicted with reasonable certainty by performing prenatal genetic analysis. However, such associations were primarily demonstrated in European and American, mostly in the Caucasians (Krone et al., 2013; Stikkelbroeck et al., 2003; Wilson et al., 2007). A comprehensive genotype–phenotype correlations have not been well established in Asian, especially in Chinese patients.

Thus, we enrolled a Chinese 21OHD cohort to investigate the clinical, biochemical, and genetic characteristics of this disorder. Detailed clinical data were collected and we found that serum FSH and LH levels could be a promising biomarker for predicting the severity of the disease. Moreover, we identified four novel mutations and evaluated their functional role using 21‐hydroxylase activity assays and in silico predictions of protein structure. Furthermore, we analyzed genotype–phenotype correlations in both the cohort and 487 Chinese 21OHD patients reported previously to accumulate valuable data on the features of 21OHD in Asian. Our research may assist in the understanding of phenotypic variability and provide useful insight into the characteristics of 21OHD as well as prenatal diagnosis for this disease.

## 2 PATIENTS AND METHODS

### 2.1 Ethical Compliance

This study was approved by the Ethics Committee of the Shandong Provincial Hospital. A written informed consent was obtained from all patients or their guardians.

### 2.2 Patients

From 2012 to 2017, a total number of 72 unrelated patients who were diagnosed as 21OHD and treated in Shandong Provincial Hospital were studied. In order to facilitate the diagnosis of the patients, plasma levels of 17OHP, testosterone (T), corticotropin releasing hormone (ACTH), cortisol (COR), prolactin (PRL), estradiol (E2), PRG, follicle‐stimulating hormone (FSH), and luteinizing hormone (LH) were determined at diagnosis by an electrochemiluminescence immunoassay (Roche, Switzerland). Serum samples were analyzed on the Cobas e 601 automated analyzer (Roche). Specific anti‐human monoclonal antibodies were used to exclude the cross‐reactions with other hormones. The diagnoses of the patients were confirmed by molecular genetic testing. The phenotype classification was based on clinical symptoms and hormonal level (Stikkelbroeck et al., 2003). In addition, we searched the NCBI Pubmed (https://www.ncbi.nlm.nih.gov/pubmed) and Wanfang (http://www.wanfangdata.com.cn/index.html) database using the term “CYP21A2 and Chinese/China.” Finally, we collected the clinical and hereditary data of 415 Chinese 21OHD patients reported previously. The genotype and phenotype information of these 487 patients were summarized and reviewed (Chan et al., 2011; Li, Luo, & Maimaiti, 2016; Liao, Zhang, & Gu, 2003; Ma et al., 2014; Wang, Gu, et al., 2016; Wang, Yu, et al., 2016; Yu et al., 2011; Yue et al., 2017; Zheng et al., 2014).

### 2.3 Mutation analysis of *CYP21A2*

Genomic DNA was extracted from peripheral blood leukocytes sampled from all the patients using a genomic DNA kit (TIANGEN BIOTECH, Beijing). Specific amplification of *CYP21A2* by PCR was performed as previously described (Menassa et al., 2008), followed by sequencing of the entire *CYP21A2* gene. The resulting sequences were compared to the corresponding wild‐type sequences of *CYP21A2* using the AutoAssembler software (version 2.0; Perkin Elmer). *CYP21A2 *mutations were named according to Human Genome Variation Society nomenclature guideline (http://www.hgvs.org/mutnomen) by using RefSeq sequence (NM_000500.7). One hundred normal human samples were sequenced to exclude that new genetic variations found in the present research were polymorphism.

### 2.4 Molecular modeling

The effect of novel mutations on the structure of the *CYP21A2* protein (the deletion of residues 199–485, residue 321, residues 459–464, and mutation His393Gln) was evaluated by studying the 4Y8W structure of human cytochrome P450 21A2 using PyMoL 1.7.6 (DeLano Scientific, LLC, Portland, USA) (Pallan et al., 2015). Multiple amino acid sequences of CYP21 orthologs from species including human (P08686), pig (P15540), bovine (P00191), dog (Q8WNW0), cat (Q2LA60), rat (Q64562), and mouse (P03940) were obtained from the Uniprot database.

### 2.5 Construction of plasmids and site‐directed mutagenesis

The human full‐length *CYP21A2* cDNA was cloned into the pcDNA 3.1 vector, resulting in the pcDNA3.1‐CYP21A2‐WT construct. Four novel mutations were introduced by site‐directed mutagenesis using the corresponding mutagenesis primers. Primers used for mutagenesis were listed in Table S1. The plasmids were named according to the designed mutations pcDNA3.1‐CYP21A2‐L199X, E321del, H393Q, L459_P464del, I173N, V282L, P454S, and pcDNA3‐CYP21A2‐P483S. In addition to the novel mutations, four control mutations were used for validation: I173N, V282L, P454S, and P483S with about 4.4%, 18%, 24%–38%, and 54%–61% residual 21‐hydroxylase activity (Tardy et al., 2010). These controls were used for standard normalization of the enzyme activity assay and comparison with others in the literature. All the constructed plasmids were verified by direct sequencing to check for the expected mutation and exclude additional sequence aberrations.

### 2.6 Cells and transfection

COS‐7 cell line obtained from the American Type Culture Collection (ATCC) was maintained in DMEM medium (Gibco, CA), supplemented with 10% inactivated fetal bovine serum (FBS) (Gibco) in a humidified cell incubator with an atmosphere of 5% CO_2_ at 37℃. A total number of 5 × 10^5^ cells (50%–70% confluence) were transiently transfected with 2 µg related *CYP21A2* plasmid using Lipofectamine 2000 according to the manufacturer's protocols (Invitrogen, Carlsbad, CA, USA). A GFP‐labeled plasmid was also cotransfected into the cells and the fluorescence intensity was recorded to verify the transfection efficiency.

### 2.7 Assays of enzyme activity

The activity of 21‐hydroxylase in intact COS‐7 cells was determined 48 h after transfection. The cells were incubated for 1 hr at 37℃ with 500 µl DMEM medium containing 0.5 µCi ^3^H‐labeled substrate (PRG or 17OHP), 2 µmol/L unlabeled substrate, and 8 mmol/L nicotinamide adenine dinucleotide phosphate reduced (NADPH, Sigma‐Aldrich, St. Louis, MO).

After incubation, steroids were extracted from the culture medium with methanol/ethylacetate (1:1, vol/vol), evaporated to dryness, and dissolved in methanol. The steroids were separated by thin‐layer chromatography (TLC) using chloroform/acetone (7:3, vol/vol) and the radioactivity was measured by liquid scintillation counting. Cells were trypsinized and lysed in lysis buffer, protein concentrations of the homogenized lysates were measured using a BCA protein assay kit (Thermo Fisher Scientific, Shanghai, China).

21‐Hydroxylase activity was expressed as a percentage of conversion grade, taking the activity of cells expressing the CYP21A2‐WT protein as 100% after correction for total protein and for the activity of cells transfected with the empty pcDNA3.1 plasmid. Enzymatic activity was calculated using the Graph Pad Prism software (version 4.0, GraphPad Software Inc., San Diego, CA).

To ensure similar amount of *CYP21A2* expression in transfected cells, western blotting analysis was performed using rabbit polyclonal antibodies against human *CYP21A2* as primary antibody (Sigma‐Aldrich, Saint Louis, USA) and anti‐rabbit IgG (Cell Signaling Technology, Danvers, MA) as the secondary antibody.

All these experiments were accomplished in the Biomedical Isotope Research Center of Shandong University.

### 2.8 Correlation between genotype and phenotype

The 72 patients were divided into five groups according to their genotype. The categorization based on the established in vitro residual enzyme activity and on the assumption that the mildest mutation determines the phenotype in compound heterozygotes. Group null consisted of mutations abolishing enzyme activity that associated with SW form, as large genetic lesion (large gene deletion and conversion), E6 cluster (I237N, V238E, M240L), G111Vfs*21, Q319X, and R357W. Group A contained mutation I2G (IVS2‐13A/C > G) that had 0%–1% residual enzyme activity, which is typically associated with SW CAH. Group B consisted mainly of the mutation I173N with a very low residual 21‐hydroxylase activity (1%–10%), which is predicted to have SV type. Group C included patients with promoter conversion (c.‐126C > T, C.‐113G > A), A16T, P31L, S269T, V282L, R484Q, and R342W mutations (5%–75% residual enzymatic activity), which may resulted to the mildest NC type (Asanuma et al., 1999; Dolzan et al., 2003; Lajic et al., 1997; Speiser & New, 1987; Stikkelbroeck et al., 2003).

All the genotypes of 487 Chinese patients ever reported were analyzed according to the order of the severity of the genotypes, similar to the above‐mentioned categorization.

### 2.9 Statistical analysis

Patients were stratified on the basis of their genotypes into mutation groups. Quantitative data are presented as the mean ± *SD*. Continuous variables were analyzed using one‐way ANOVA, followed by the post hoc comparisons using Tukey's test. Median ± IQR and Kruskal–Wallis tests were used when the assumptions of Gaussian distributions were not met. Receiver operating characteristic (ROC) curve analysis was performed to assess the predicting value of FSH and LH. All statistical analyses were performed using SPSS 18.0 software (SPSS Inc., Chicago, USA), and a *p* < 0.05 was considered statistically significant.

## 3 RESULTS

### 3.1 Clinical and biological features of patients

Detailed clinical information of each patient was listed in Table [Link], [Link]. The patients consisted of 37 males and 35 females. The average diagnosed age was 3.5 ± 7.6 years (range 0–29). Among them, 47 patients (65.3%) were diagnosed as SW phenotype, 11 (15.3%) were SV type, and 14 (19.4%) were NC type. Hyperpigmentation was the most common symptom (62.5%). Other common symptoms were clitoromegaly in 37.5% and advanced penile growth in 13.9% of the patients. Fifty‐six patients were infants when diagnosed, among them, milk regurgitation (24 in 56, 42.9%) and no weight increase (7 in 56, 12.5%) were the most common symptoms.

**Figure 1:**
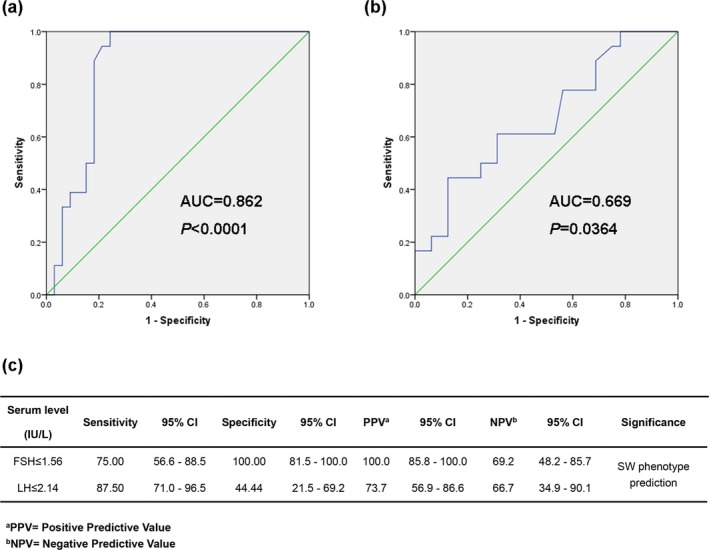
The diagnostic significance of serum FSH and LH levels in CAH patients. (a‐b) The ROC curves showed strong separation between the severe phenotype and mild phenotype using serum FSH level and LH level, with an AUC of 0.862 (*p* < 0.0001) and 0.669 (*p* = 0.0364), respectively. (c) Sensitivity, specificity, and positive and negative predictive values for the prediction of SW phenotype using serum FSH and LH level

The mean value of FSH (1.82 ± 3.25 IU/L) and LH (0.56 ± 1.88 IU/L) were under the lower limit of the normal range of FSH (5–40 IU/L) and LH (5–25 IU/L). To determine the predicting value of serum FSH and LH levels in disease severity, we constructed ROC curves and calculated the area under the curve (AUC) in 51 patients who have measured the hormonal levels. The ROC curves suggested that the AUC value of FSH for the prediction of the severe SW phenotype was up to 0.86 (Figure 1a, CI (95%): 0.74–0.94, *p* < 0.001), with an estimated sensitivity and specificity of 75.00% and 100.00%, respectively (Figure 1c). While the AUC value of LH for predicting the SW phenotype was 0.67 (Figure 1b, CI (95%): 0.52–0.80, *p* = 0.036), with an estimated sensitivity and specificity of 87.50% and 44.44%, respectively (Figure 1c).

### 3.2 Mutational analysis of the *CYP21A2* gene

**Figure 2:**
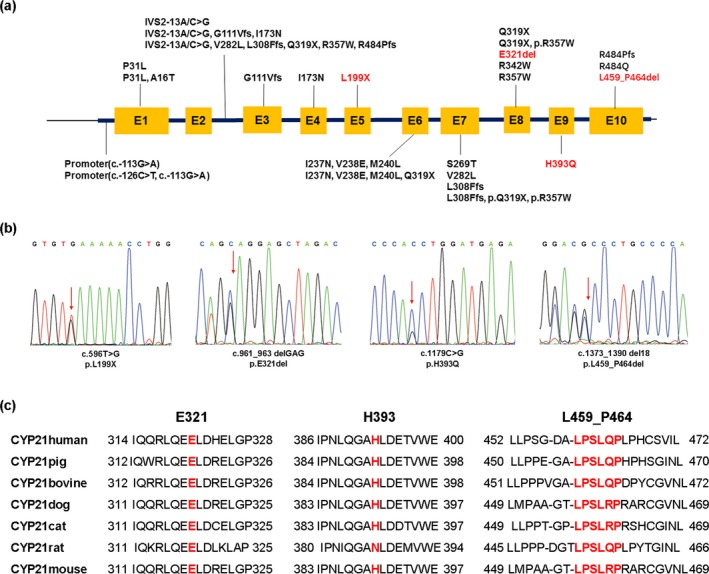
The position, sequence of the mutations. (a) Schematic presentation of *CYP21A2* gene and localization of mutations detected in the Chinese population of CAH patients, including the four novel mutation shown in red bold. (b) Four novel mutations including c.596T > G, c.961_963 delGAG, c.1179C > G, and c.1373_1390del18 which is all depicted by a red arrow in the DNA sequences. (c) Multiple amino acid alignment of CYP21 orthologs from the Uniprot database including sequences from species in the following order: human (P08686), pig (P15540), bovine (P00191), dog (Q8WNW0), cat (Q2LA60), rat (Q64562), and mouse (P03940). The mutant amino acids of human *CYP21A2 *gene and corresponding residues of aligned sequences are shown in red bold

*CYP21A2* mutational analysis was performed in 72 patients with 21OHD. The overall detection rate of mutation was 98% (141/144). Only one mutant allele was detected in three patients. Then, 141 unrelated alleles were studied, which revealed 25 mutations and 36 genotypes (Figure 2a, Table 1). Homozygosity was found in 15 of the 72 patients (21%), while compound heterozygosity was present in 54 patients (75%). Only one pathogenic allele was identified in 3 of the 72 patients (4%). The most frequently mutations in the present research were I2G (33%), Large lesion (21%), I173N (17%) and Q319X (7%).

**Table 1:** Distribution of CYP21A2 mutations in 72 patients with 21‐hydroxylase deficiency

Location	Change at the DNA level (c.)	Change at the protein level (p.)	Mutated allele	
			*n*	%
	Gene deletions/duplications/rearrangements	Large lesion	29	20.6
5'UTR	c.−113G > A	Promoter	1	0.7
5'UTR	c.−126C > T, c.−113G > A	Promoter	1	0.7
Exon 1	c.92C > T	p.P31L	2	1.4
Exon 1	c.92C > T, c.46G > A	p.P31L, p.A16T	1	0.7
Intron 2	c.290−13A/C > G	I2G	46	32.6
Intron 2, exon 3, exon 4	c.290−13A/C > G, c.332−339del8, c.518 T > A	I2G, p.G111fs, p.I173N	1	0.7
Intron 2, exon 7, exon 8, exon 10	c.290−13A/C > G, c.844G > T, c.923dupT, c.955C > T, c.1069C > T, c.1450dupC	I2G, p.V282L, p.L308fs, p.Q319X, p.R357W, p.R484Pfs	1	0.7
Exon 3	c.332_339del8	p.G111fs	2	1.4
Exon 4	c.518T > A	p.I173N	24	17.0
Exon 6	c.710T > A,c.713T > A,c.719T > A	E6 Cluster	1	0.7
Exon 6, exon 8	c.710T > A,c.713T > A,c.719T > A, c.955C > T	E6 Cluster, p.Q319X	1	0.7
Exon 7	c.806 G > C	p.S269T	3	2.1
Exon 7	c.844G > T	p.V282L	1	0.7
Exon 7	c.923 dupT	p.L308Ffs	2	1.4
Exon 7, exon 8	c.923dupT, c.955C > T, c.1069C > T	p.L308Ffs, p.Q319X, p.R357W	1	0.7
Exon 8	c.955C > T	p.Q319X	10	7.1
Exon 8	c.955C > T, c.1069C > T	p.Q319X, p.R357W	2	1.4
Exon 8	c.1024C > T	p.R342W	1	0.7
Exon 8	c.1069C > T	p.R357W	5	3.5
Exon 10	c.1451_1452delGGinsC	p.R484Pfs	1	0.7
Exon 10	c.1451G > A	p.R484Q	1	0.7
Novel mutations				
Exon 5	C.596T > G	p.L199X	1	0.7
Exon 8	c.961_963delAGG	p.E321del	1	0.7
Exon 9	c.1179C > G	P.H393Q	1	0.7
Exon 10	c.1373_1390 del18	p.L459_P464del	1	0.7

Direct sequencing of the entire *CYP21A2* gene identified four novel compound heterozygous mutations, including one missense mutation (c.1179C > G, p.H393Q), one nonsense mutation (c.596T > G, p.L199X), and two small deletions (c.961_963delAGG, p.E321del and c.1373_1390 del18, p.L459_P464del) (Figure 2b). The residue 321,393 and residues 459–464 were highly conserved in CYP21 of mammals (Figure 2c). These novel mutations affected the highly conserved domains among vertebrate orthologs, while the nonsense mutation c.596 T > G leaded to a truncated protein.

### 3.3 In silico structure analysis

**Figure 3:**
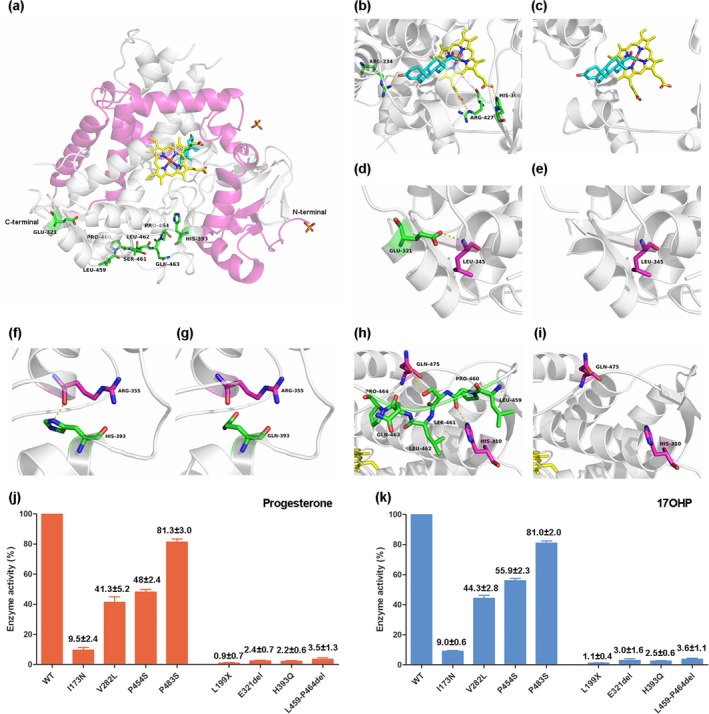
The in silico predictions of the four novel mutations and the enzymatic activities of *CYP21A2* mutant proteins. (a) Overall structure of the CYP21A2 protein with heme in yellow, progesterone as a substrate in cyan. The residue GLU‐321, HIS‐393 and residues LEU459‐PRO464 are shown in green. The residues 1–188 are shown in rose red and the deleted residues 199–485 are shown in gray. (b) CYP21A2 wt: The yellow dotted line shows the electrostatic interaction between the side chain of Arg‐234 (green) and the progesterone substrate molecule (cyan), and the side chain of His‐366 and Arg‐427 (green) and the heme (yellow). (c) CYP21A2 Leu199X: In the protein, there is no electrostatic interaction between residues and the substrate progesterone (cyan) and heme (yellow). (d) CYP21A2 wt: The yellow dotted lines show the electrostatic interactions between the side chain of Glu‐321 (green) and Leu‐345 (rose red). (e) CYP21A2 Glu321del: In the mutant protein, the electrostatic interaction between the residue and Leu‐345 (rose red) is lost. (f) CYP21A2 wt: The yellow dotted lines show the electrostatic interactions between the side chain of His‐393 (green) and Arg‐355 (rose red). (g) CYP21A2 His393Gln: In the mutant protein, the electrostatic interaction between Gln‐393 (green) and Leu‐345 (rose red) is lost. (h) CYP21A2 wt: The yellow dotted lines show the electrostatic interactions between the side chain of Ser‐461 (green) and Gln‐475 (rose red), and between the residues Pro‐460 (green) and His‐310 (rose red). (i) CYP21A2 459‐464del: In the protein, the electrostatic interaction between the residues and Gln‐475 and His‐310 (rose red) is lost. (j, k) Residual activity of the different mutant proteins in COS7 cells. Activities are expressed as a percentage of wild‐type activity (WT), which is defined as 100%. Conversion values are shown for the two natural substrates (progesterone and 17OHP) to their corresponding products. The bars represent the mean ± 1SD (*n* = 3)

As is shown in Figure 3a, in silico analysis revealed that steroid 21‐hydroxylase featured 13 α‐helices and a total of nine β‐strands. The latter folded into an N‐terminal β‐sheet domain (β1–β7) and a C‐terminal sheet (β8–β9). The structural impacts of the residues 199–485, residue 321, and residues 459–464 deletions, as well as His393Gln mutation were further investigated by studying the human cytochrome P450 21A2 structure using PyMoL 1.7.6. The deletion of the residues at the position 199–485 leaded to loss of electrostatic interactions with the substrate progesterone and heme (Figure 3b,c), while the deletion of glutamic acid at position 321 disturbed the interaction with Leu‐345 (Figure 3d,e). In addition, a change to glutamine at position 393 influenced the interaction with Arg‐355 (Figure 3f,g). The removal of the residues at the positions 459–464 resulted in loss of electrostatic interactions with the residues His‐310 and Gln‐475 (Figure 3h,i).

### 3.4 In vitro functional analysis

In order to characterize the effect of the four novel *CYP21A2 *mutations on the CYP21 activity, we detected the in vitro residual activities of these mutations and compared the results to the residual activities of four well‐known reference mutations (p.I173N, p.V282L, p.P454S, and p.P483S). Enzyme activities toward the two natural substrates, PRG and 17OHP, were evaluated in intact COS‐7 cells, and relative enzyme activities were present in histogram (Figure 3j,k). The activity of the normal protein (WT) was defined as 100%, and consistent with previous research, conversion of PRG and 17OHP for p.I173N, p.V282L, p.P454S, and p.P483S were gradually increased. All the four novel mutations showed a significantly reduced CYP21 activity of less than 5% compared with the wild type, both for the PRG and 17OHP. Especially for the mutation L199X, which showed a conversion rate of lower than 1% for PRG to deoxycorticosterone.

### 3.5 Correlation between genotype and phenotype

In this study, 72 patients were categorized into four groups according to phenotype and residual enzyme activity for further analysis: group null 12 (seven males, five females), group A 26 (19 males, seven females), group B 16 (three males, 13 females), and group C 11 (four males, seven females). The rest were patients with novel mutations and one mutation (four males, three females) (Table 2). In group null, all 12 patients (100%) had the classic SW form as expected. In group A, 23 of the 26 patients (88.5%) had the anticipated SW form, and only 1 patient (3.8%) was diagnosed with the SV form and 2 (7.7%) were diagnosed with the NC form. There were 5 (31.3%), 6 (37.5%), and 5 (31.3%) patients of 16 patients in group B exhibited SW, SV, and NC phenotypes, and 2 (20%), 4(40%), and 4(40%) of 10 patients in group C, respectively. The rest seven patients included three with only one pathogenic mutation and four with the four novel mutations. Combined with the clinical manifestations and the results of in vitro enzyme activity studies, all the four novel mutations were associated with SW phenotype, and the related patients should be classified to group A. Meanwhile, the three heterozygous patients were all diagnosed with the NC form. The diagnosed age and hormone data of the 65 patients were shown for the total cohort and for males and females in 3. The diagnosed age remarkable rose from group null to C (*p* < 0.05). There were statistical difference in the variations of 17OHP, COR, E2, FSH, and LH between the four groups (*p* < 0.05). Among them, the 17OHP levels markedly decreased from group null to C in both male and female patients. Meanwhile, the levels of COR and E2 increased significantly from group null to C in the entire patients. The serum levels of PRG and PRL differed in the female groups significantly (*p* < 0.05), but no significant difference was shown in the male groups. We also noticed a trend toward higher expression of T, ACTH, and PRL in patients with predicted SW CAH (groups null and A) than in the patients in groups related to SV CAH (group B) or NC CAH (group C).

**Table 2:** Genotype and phenotype in 72 patients with 21‐hydroxylase deficiency

Genotype	Allele 1	Allele 2	Phenotype					
			Males			Females		
			SW	SV	NC	SW	SV	NC
Group null	Q319X	Large lesion	2			4		
	G111Vfs*21	Large lesion	1					
	I2G, G111Vfs*21, I173N	Large lesion	1					
	R357W	Large lesion	1					
	E6 cluster, Q319X	Large lesion	1					
	Q319X	R357W	1					
	E6 cluster	Large lesion				1		
Group A	I2G	I2G, V282L, L308Ffs*6, Q319X, R357W, R484Pfs*40	1					
	I2G	I2G	7	1		2		
	I2G	Q319X, R357W	2					
	I2G	Large lesion	3			1		1
	I2G	R357W	2				1	
	I2G	Q319X	2			1		
	I2G	G111Vfs*21	1					
	I2G	R484Pfs*40				1		
Group B	I173N	Large lesion	2		1	2		
	I173N	I2G					4	1
	I173N	Q319X					1	
	I173N	I173N				1	1	3
Group C	S269T	L308Ffs*6	1					
	P31L, A16T	I2G	1					
	V282L	I2G		1				
	P31L	Large lesion			1		1	
	Promoter（c.−126C > T, c.−113G > A）	I2G						1
	R484Q	I2G						1
	S269T	Large lesion					1	
	R342W	Large lesion					1	
	Promoter（c.−113G > A）	Large lesion						1
Novel mutations/one mutation	H393Q	Large lesion	1					
	E321del	L308Ffs*6	1					
	L199X	I2G				1		
	L459_P464del	I173N				1		
	Normal	I173N						1
	Normal	Large lesion			1			
	Normal	Large lesion			1			
Total			32	2	4	15	10	9

**Table 3:** Hormone changes in the four groups based on their genotypes

	Gender	Genotype group^a^				*p* value
		Null (*n* = 12)	A (*n* = 26)	B (*n* = 16)	C (*n* = 11)	
Age at diagnosis (month)	All	**0.61 ± 0.73**	**14.38 ± 47.54**	**76.31 ± 102.97**	**93.29 ± 129.26**	**0.0077**
17OHP (nmol/L)	All	**551.34 ± 175.63**	**406.02 ± 185.44**	**137.27 ± 62.52**	**47.20 ± 20.71**	**0.0001**
	Male	**632.83 ± 103.13**	**428.06 ± 190.90**	**125.73 ± 78.68**	**44.40 ± 12.23**	**0.0001**
	Female	**429.10 ± 190.40**	**312.33 ± 121.67**	**141.11 ± 55.58**	**48.40 ± 23.31**	**0.0001**
Testosterone (ng/ml）	All	2.26 ± 2.01	1.96 ± 1.69	1.75 ± 1.56	1.14 ± 0.94	NS
	Male	1.57 ± 0.84	2.20 ± 1.84	1.35 ± 0.22	0.85 ± 0.46	NS
	Female	3.46 ± 2.77	1.22 ± 0.69	1.87 ± 1.75	1.33 ± 1.11	NS
ACTH (pg/ml)	All	148.71 ± 102.28	138.28 ± 115.74	125.81 ± 55.13	94.65 ± 98.12	NS
	Male	143.58 ± 107.01	159.58 ± 123.81	157.7 ± 22.91	65.41 ± 31.64	NS
	Female	157.70 ± 92.74	70.10 ± 33.30	117.11 ± 58.07	111.37 ± 117.43	NS
COR (nmol/L)	All	**190.23 ± 126.46**	**211.43 ± 123.23**	**225.74 ± 118.11**	**339.34 ± 150.88**	**0.0432**
	Male	196.78 ± 142.66	188.94 ± 132.22	168.6 ± 22.08	385.98 ± 122.13	NS (0.086)
	Female	178.78 ± 90.32	283.38 ± 32.23	241.33 ± 128.41	312.69 ± 159.05	NS
PRL (ng/ml)	All	65.55 ± 35.04	56.47 ± 36.36	41.20 ± 23.96	13.67 ± 17.71	NS (0.092)
	Male	53.50 ± 33.24	59.66 ± 37.08	54.70 ± 14.31	46.52 ± 16.01	NS
	Female	**83.62 ± 29.47**	**46.12 ± 31.79**	**36.13 ± 24.87**	**19.78 ± 6.44**	**0.0191**
E2 (pg/ml)	All	**12.71 ± 12.27**	**21.39 ± 18.09**	**35.17 ± 18.47**	**48.69 ± 45.27**	**0.0106**
	Male	13.75 ± 11.96	21.84 ± 18.37	34.93 ± 5.69	25.13 ± 3.02	NS
	Female	10.89 ± 12.59	19.61 ± 16.81	35.25 ± 21.08	64.39 ± 52.85	NS
PRG (ng/ml)	All	21.23 ± 15.89	21.35 ± 18.92	21.77 ± 11.21	12.18 ± 17.31	NS
	Male	15.17 ± 11.90	21.98 ± 20.75	16.47 ± 8.04	21.81 ± 23.56	NS
	Female	**30.31 ± 16.78**	**19.46 ± 11.64**	**23.54 ± 11.56**	**5.76 ± 5.12**	**0.0293**
FSH (IU/L)	All	**0.35 ± 1.05**	**0.99 ± 1.64**	**3.14 ± 1.38**	**4.07 ± 4.79**	**<0.001**
	Male	0.10 ± 0.49	0.99 ± 1.46	2.54 ± 0.00	1.95 ± 0.20	NS (0.0568)
	Female	0.91 ± 1.50	1.23 ± 3.26	3.30 ± 1.49	5.40 ± 1.10	NS(0.0535)
LH (IU/L)	All	**0.09 ± 1.13**	**0.39 ± 0.51**	**1.22 ± 1.40**	**2.98 ± 3.15**	**0.0122**
	Male	**0.09 ± 0.26**	**0.54 ± 0.72**	**2.42 ± 0.00**	**2.98 ± 0.21**	**0.0465**
	Female	0.74 ± 1.51	0.28 ± 0.16	1.12 ± 0.95	3.60 ± 6.11	NS (0.1071)

a Seven patients (four with novel mutations and three with one mutation) were excluded. Sixty‐five patients were categorized into four groups according to phenotype and residual enzyme activity for further analysis: group null 12 (7 males, 5 females), group A 26 (19 males, 7 females), group B 16 (3 males, 13 females), and group C 11 (4 males, 7 females).

Values with statistical significance showed in bold.

**Figure 4:**
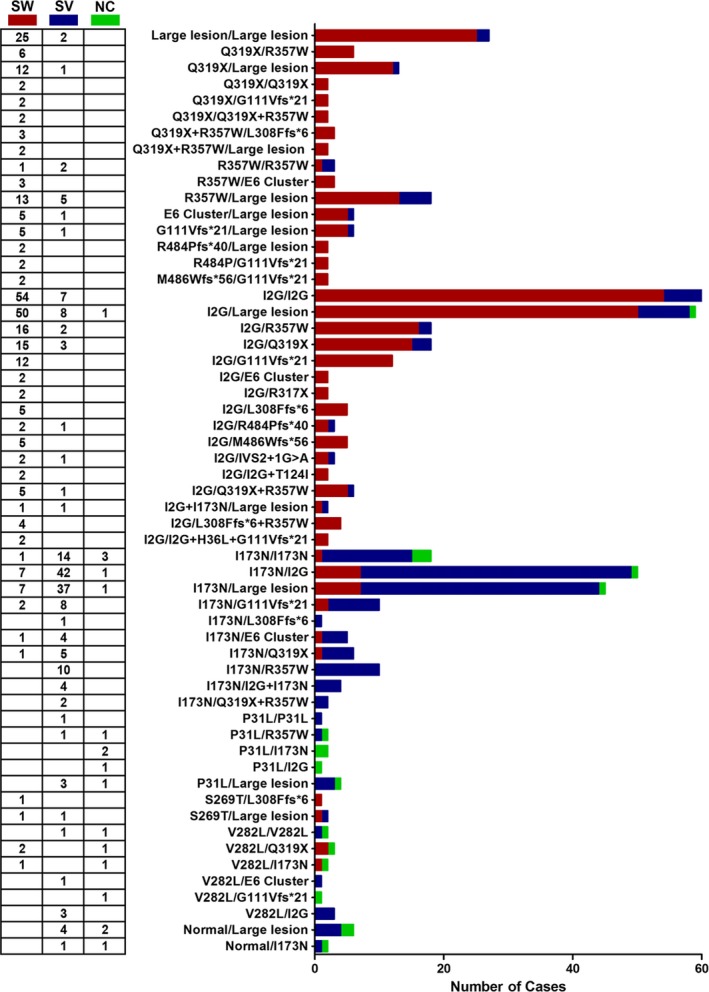
Frequency of *CYP21A2* genotypes in 487 CAH patients of China. The number of CAH patients with each of the *CYP21A2* genotypes (SW, red; SV, blue; NC, green) is shown

In order to further verify the genotype–phenotype correlations of this disease, we summarized all the previously reported Chinese 21OHD patients in Figure 4. The genotypes were listed from top to bottom according to severity. Generally, disease severity can be predicted with the genotypes. It was obviously that the phenotypes changed from the severe SW form (*in red*) in the top to the less severe SV and NC forms in the bottom (*in blue and green*). The most common genotypes in Chinese population were I2G/I2G (12.5%), I2G/Large lesion (12.1%), I173N/I2G (10.3%), and I173N/Large lesion (9.2%). While the most frequently mutations were I2G (33.9%, 327 in 966 alleles), Large lesion (22.7%, 219 in 966 alleles), I173N (18.7%, 181 in 966 alleles), and Q319X (13.3%, 128 in 966 alleles). A direct genotype–phenotype correlation was found with several mutations. The SW form of CAH is prominent in deletion or intronic splice mutations, namely I2G/I2G (18.6%), I2G/Large lesion (17.2%), and Large lesion/Large lesion (8.6%). In contrast, SV CAH was observed most frequently in I173N/I2G (23.5%) and I173N/Large lesion (20.7%) mutations. We identified in Chinese population that a combination of any of the following mutations, Q319X, R357W, E6 cluster (I237N, V238E, M240L), and G111Vfs*21 will almost invariably result in SW form. Furthermore, certain mutations can cause different phenotypes. Our data showed that SV CAH occurs in 84.1% of the patients who carried the I172N mutation. Approximately 12.6% of these patients present with SW CAH and 3.31% with NC CAH. Moreover, the most frequent mutation, I2G, results primarily 70.1% in SW, while 27.9% patients present with SV, 1.1% with NC (Table 3).

## 4 DISCUSSION

For the first time, we analyzed the related sex hormones of the 21OHD patients. The serum levels of E2, LH, and FSH differed with statistical significance among the four groups. It is well‐known that 21OHD could lead to the excessive androgen secretion, which had a negative feedback effect on the hypothalamic–pituitary–gonadal axis (HPGA). Then, gonadotropin‐releasing hormone (GnRH), LH and FSH secretion are decreased, causing hypogonadotropic hypogonadism and gonadal dysgenesis (Turcu & Auchus, 2015). As upstream hormones in the HPGA, LH and FSH vary sensitively when androgen and estrogen changes. Since one important therapeutic goal for the treatment of CAH is to normalize sex hormones, the optimal glucocorticoid and mineralocorticoid dose should suppress 17OHP and its metabolites and maintains sex hormone concentrations in the age and sex‐specific normal range at the same time (Webb & Krone, 2015). Similar to the function of TSH in the thyroid disease, FSH and LH might be served as a potential marker for the prediction of disease severity. Meanwhile, they could be valuable in evaluating therapeutic outcome or guiding the clinical treatment of patients with 21OHD. As decreased expression of E2 and PRG (significantly in female) in the severe groups may result from decreased LH and FSH secretion. All the hormone data still needs to be verified in a larger cohort.

Four novel mutations of *CYP21A2* were detected in four of our patients with SW form. The nonsense mutation c.596 T > G leaded to a truncated protein p.L199X. In vitro studies of it confirmed a very low residual activity about 0.9% for the conversion of PRG and 1.1% for the conversion of 17OHP. The missing residues 199–485 contains nine α‐helices (F, F’, G, H, I, J, K, L, and M), as well as β4‐β9 strands (Robins, Carlsson, Sunnerhagen, Wedell, & Persson, 2006). The deletion of the nine α‐helices and the six β‐strand would directly disturb the electrostatic interactions and hydrophobic interactions with the heme and progesterone, resulting in absent enzyme activity.

The new p.E321del (c.961_963delAGG) mutation showed a residual activity of 2.4% for the conversion of PRG and 3.0% for the conversion of 17OHP. The residue Glu‐321 was highly conserved in CYP21 enzymes of different species and localized in J helix. This helix located in the surface of the protein. The deletion of the polar glutamic acid would disturb the interaction with Leu‐345, which was localized in the highly conserved K helix, indirectly interfering with heme binding and consequently abolishing *CYP21A2 *protein activity.

The residue His‐393 was located in the highly conserved L helix, and formed electrostatic interaction with the residue Arg‐355, which belonged to conserved K helix. The substitution of the polar amino acid histidine for neutral glutamine in the protein core would lead to a severe loss of enzyme function. Our in vitro studies confirmed the mutation c.1179C > G (p.H393Q) about 2.2% for the conversion of PRG and 2.5% for the conversion of 17OHP.

In vitro analysis of the p.L459_P464del confirmed a residual activity of 3.5% for the conversion of PRG and 3.6% for the conversion of 17OHP. The residues 459–464 were conserved in CYP21 in many different species. They were located in the β8‐β9 connecting loop region, and formed electrostatic interaction with the residue Gln‐475, which was also localized in the β8‐β9 connecting loop, and the residue His‐310, which belonged to I helix. The deletion of the six residues would disturb the electrostatic interactions with His‐310 and Gln‐475, and perturb hydrophobic interactions with the residues around them, indirectly interfering with heme and progesterone substrate molecule binding and consequently influencing the enzyme activity.

In the cohort of the 72 patients, we found a high correlation between genotype and phenotype in the severe groups (group null, A). All the patients (100%) in group null had the expected SW phenotype. In addition, 88.5% of the patients in group A had the SW phenotype as anticipated. These results were all in accordance with the previous research (Wedell, Thilen, Ritzen, Stengler, & Luthman, 1994). Our genotype–phenotype correlation was poor for groups B and C. In group B, 5 (31.3%) patients had the predicted SV form. In group C, only 4 (36.4%) of the 11 patients had the predicted NC form. The noncorrelation of groups B and C in our research might be resulted from the small sample size, and this phenotypic variability was partially in accordance with previous reports showing that phenotypes in the two groups were quite heterogeneous. The lack of genotype‐phenotype correlation on groups B and C might also be due to the lack of clear clinical and laboratorial criteria previously established for the CAH forms, suggesting a weakness of the study. Besides, the mutation I173N caused a wide range of enzymatic activity possibly contributing to phenotypic variability in group B associated with SV type (Jaaskelainen, Levo, Voutilainen, & Partanen, 1997; Krone, Braun, Roscher, Knorr, & Schwarz, 2000).

We also performed genotype‐phenotype analysis in 487 Chinese 21OHD ever reported. Compared with the previous report, the frequency of V282L (1.4%) detected in Chinese population was much lower than that detected in all the countries (23.9%), but similar to Dutch population (2.2%). The presence of the V282L mutation may exist ethnic difference. On the other hand, this result may be due to the low detectable rate of NC CAH in China. Because the V282L mutation leaded to a mild enzyme deficiency was usually observed in the NC phenotype. Meanwhile, the frequency of I2G, I173N and Q319X (33.9%, 18.7%, and 13.3%) in Chinese patients was much higher than that in all the populations (22.9%, 8.2%, and 3.5%). A striking feature of Chinese patients was that only 18 of 487 patients (3.7%) showed NC phenotype, far below the NC CAH ratio in other populations (36.3%, 545 in 1507 patients) (New et al., 2013; Wilson et al., 2007). The main reason might be that most NC are found in the young adult age and subjects enrolled in our analysis were mainly children. In general, our data is partially consistent with reports published previously of 21OHD in other countries and ethnic groups (New et al., 2013; Stikkelbroeck et al., 2003; Wilson et al., 2007). Meanwhile, diversities were found which further demonstrated the ethnic specificity of the mutations in *CYP21A2* gene.

In this study, a total number of 72 21OHD patients were enrolled. A relatively small sample size might be a limitation. We are still enrolling patients and following up this cohort.

In conclusion, our present study of Chinese CAH patients had a number of considerable findings. We suggest routine FSH and LH levels might serve as a potential marker for the prediction of disease severity before treatment. Our analysis of genotype–phenotype correlations in a large cohort provides a framework for clinicians to perform a prenatal diagnosis of CAH and to predict the general phenotype of CAH for a given genotype. Moreover, we evaluated the functional role of 4 novel mutants (p.L199X, p.E321del, p.H393Q, and p.L459_P464 del) in detail, including in vitro and in silico analysis, which can enrich the human genomic database and provide novel insights into the structure–function relationship of CYP21 and other P450 enzymes. In addition, the genotype pattern of the Asian population is possibly different from the Caucasian population and that the genotype–phenotype relationship may not follow the pattern of most Western populations, which further reinforces the importance of an adequate clinical and laboratory evaluation.

## CONFLICT OF INTEREST

The author reports no conflicts of interest in this work.
